# Role of spin-glass behavior in the formation of exotic magnetic states in GdB_6_

**DOI:** 10.1038/s41598-020-75327-8

**Published:** 2020-10-26

**Authors:** A. V. Semeno, M. A. Anisimov, A. V. Bogach, S. V. Demishev, M. I. Gilmanov, V. B. Filipov, N. Yu. Shitsevalova, V. V. Glushkov

**Affiliations:** 1 Prokhorov General Physics Institute of the Russian Academy of Sciences, Vavilov str. 38, Moscow, 119991 Russia; 2 National Research University Higher School of Economics, Myasnitskaya str. 20, Moscow, 101000 Russia; 3 Frantsevich Institute for Problems of Materials Science NAS, Krzhyzhanovsky str. 3, Kiev, 03680 Ukraine

**Keywords:** Magnetic properties and materials, Phase transitions and critical phenomena

## Abstract

Randomness and frustration are believed to be two crucial criteria for the formation of spin glass state. However, the spin freezing occurs in some well-ordered crystals below the related temperature *T*_*f*_ due to the instability of each spin state, which induces the variation of either magnetic moment value or exchange energy. Here we explore the new mechanism of the in-site originated disorder in antiferromagnets Gd_0.73_La_0.27_B_6_ and GdB_6_, which is caused by the random mutual shifts of Gd^3+^ spins from the centrally symmetrical positions in the regular cubic lattice. The universal scaling of ESR linewidth temperature dependencies to the power law *ΔH*(*T*) ~ ((*T *− *T*_*D*_)*/T*_*D*_)^*α*^ with *α* = − 1.1 ± 0.05 in the paramagnetic phase of both compounds demonstrates the identity of the origin of magnetic randomness. In Gd_0.73_La_0.27_B_6_ the resulting random spin configurations freeze at *T*_*f*_ ≈ 10.5 K where the maximum of magnetization is observed. Below *T*_*f*_ the splitting of ZFC and FC magnetization curves takes place as well as the magnetic state depends on the antecedent sample history. In the case of GdB_6_ the coherent displacement of Gd ions compete with these random shifts forming an antiferromagnetic (AFM) phase at *T*_*N*_ = 15.5 K, which prevails over the spin freezing at *T*_*f*_ ≈ 13 K, expected from the ESR data. The observation of the hysteresis of the ESR spectrum in the AFM phase suggests that its properties may be determined by the competition of two types of AFM orders, which results in formation of stable magnetic domains with nonequivalent positions of AFM Gd pairs at *T* < 10 K.

## Introduction

The discovery of spin glass (SG) freezing in the stoichiometric compound URh_2_Ge_2_^1^ gave an impetus to the active search and study of SGs without inherent disorder of spin structure although some systems of this type were previously known^2^. The key point is that the basic physical concepts assume disorder and frustration/alternation as two necessary conditions to the onset of the SG state. It turned out that in URh_2_Ge_2_ the crystallographic random-bond disorder originates from the uncontrolled interchange of Rh and Ge ions^3^ and the enhanced annealing leads the substance to AFM ordering^4^. The effect of randomness of nonmagnetic ions on the emergence of SG state was found later in some other triple intermetallic compounds^5–9^. In its turn, SG behavior in perfectly ordered lattices was revealed in two groups of systems and microscopic mechanisms of the *in-site self-originated disorder* were proposed to explain this phenomenon. In praseodymium intermetallic compounds (PrAu_2_Si_2_^10^, PrIr_2_B_2_^11^, PrRuSi_3_^12^, PrRhSn_3_^13^, Pr_3_Ir^14^) the disorder appears due to the ability of Pr ions to form two magnetic states: nonmagnetic singlet and magnetic doublet. The fluctuations between these states are mediated by the exchange coupling and the instability of Pr ion occurs in a curtain critical range of this parameter, which leads to freezing of the magnetic system to SG state^15^. Pyrochlores A_2_B_2_O_7_ is another group of materials where geometrical frustrations of the lattice favor to the emergence of different exotic states including the SG freezing in well-ordered crystals^2,16,17^. Recently the microscopic mechanism of the disorder origin in this group was proposed on the example of Y_2_Mo_2_O_7_. The displacements of Mo^4+^ ions with the corresponding formation of Mo pairs with different angles Mo–O–Mo lead to variation of exchange interactions thus preventing the long-range ordered states^18^.

**Figure 1:**
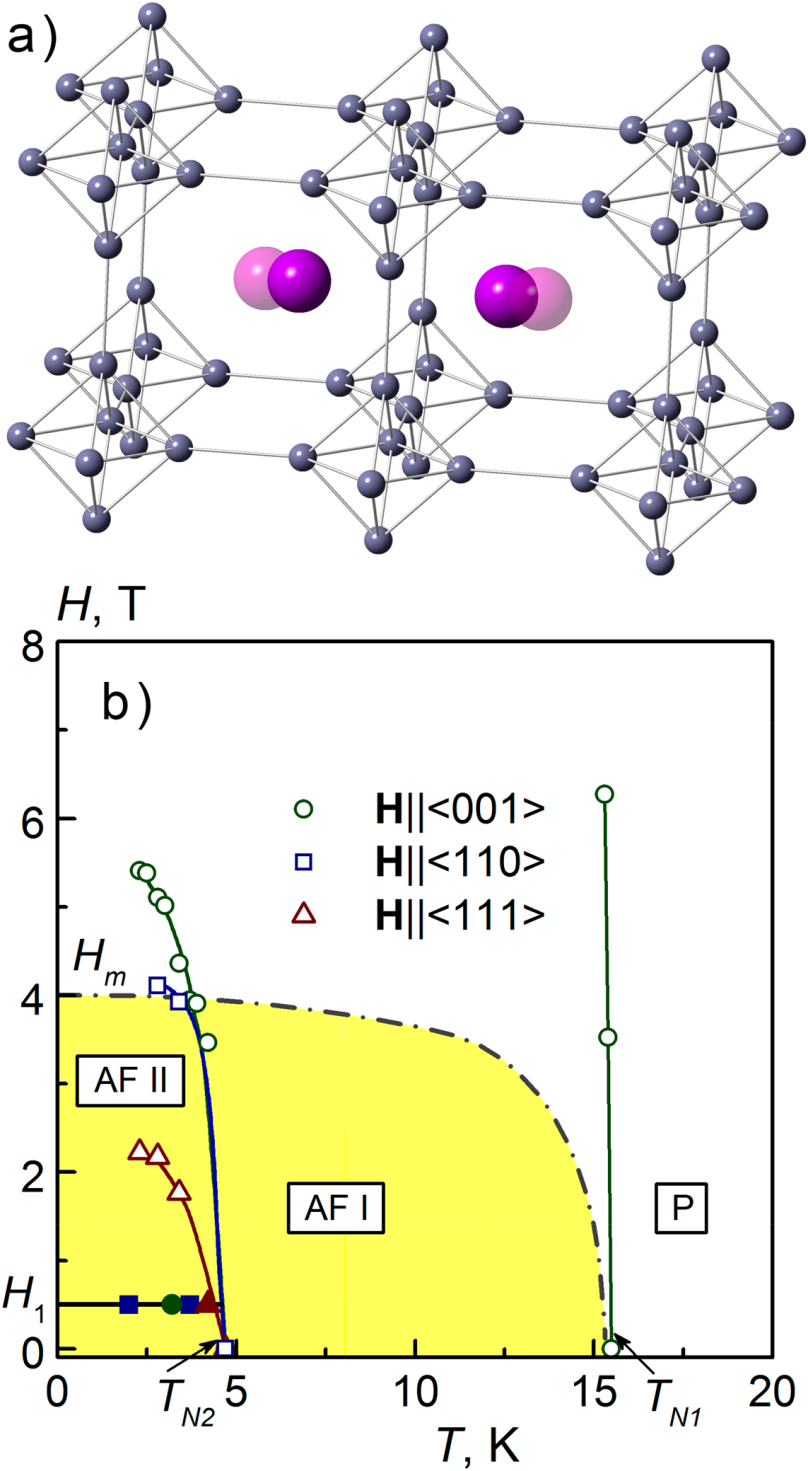
(**a**) Structure of GdB_6_ and schematic shift of Gd^3+^ ions. The concomitant shifts of boron ions are not shown. (**b**) Magnetic phase diagram of GdB_6_^33^.

In this work we explore the new mechanism of in-site originated disorder and the related SG behavior in the rich borides GdB_6_ and Gd_0.73_La_0.27_B_6_ with high symmetry cubic lattice (Pm3m − O_h_^1^). The puzzling properties of GdB_6_ are determined by the mutual shifts of neighbor Gd^3+^ ions ^8^S_7/2_(*L* = 0, *S* = 7/2) from the centrally symmetrical positions in the oversized boron lattice^19^ (Fig. 1). At high temperatures the mean square ion displacement in GdB_6_ is the highest one in the series of rare earth hexaborides (< *δ*^*2*^ > ≈1 × 10^–2^ Å^2^ at *T* = 300 K)^20^. It results in strong short range magnetic correlations in the paramagnetic (PM) phase with concomitant shift and broadening of ESR line with decreasing temperature, the deviation from Curie–Weiss behaviour starting already below *T* ~ 100 K as well as with the large ratio of Curie–Weiss parameter *Θ* = − 66 K to Neel temperature *T*_*N*_ = 15.5 K^21^. At lower temperatures *T* < 100 K the Gd^3+^ ions move in the anharmonic potential caused by their mutual magneto-elastic coupling and the interaction with carriers via the nesting of Fermi surface^22^. Such dynamic leads to softening of phonon modes^22^ inducing the growth of magnetic correlations with temperature decrease. The coherent displacement structure of Gd^3+^ ions and the concomitant AFM order become stabilized below Neel temperature *T*_*N*_ = 15.5 K via first order phase transition. The resulting structure is characterized by wavevectors [1/2,0,0] and [1/2,1/2,0] with an additional reflex [1/4,1/4,1/2] developing at temperatures *T*_*N2*_ < 10 K where new AFM2 phase appears^23–26^. The onset of the structural phase transition is accompanied by the AFM ordering with the wavevector [1/4,1/4,1/2]^27,28^. The first order type AFM transition is seen as a jump of resistivity and magnetic susceptibility at *T*_*N*_^29–31^ and also as *λ*-anomaly in the specific heat^32^. The onset of AFM2 phase is not always detected in temperature dependencies of physical parameters but is manifested by hysteretic behavior of resistivity below *T*_*N*2_^33^ (Fig. 1b).

## Results and discussion

**Figure 2:**
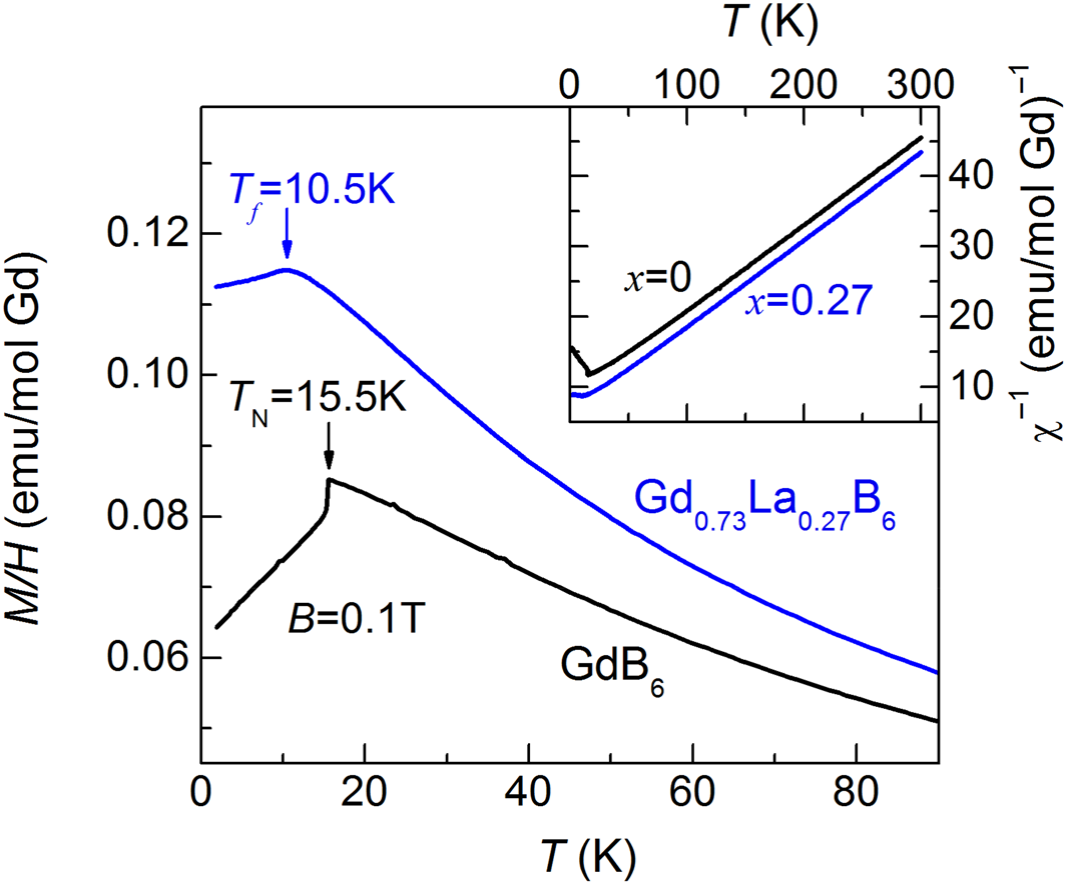
Temperature dependencies of magnetic susceptibility of Gd_0.73_La_0.27_B_6_ and GdB_6_. (inset: inverse susceptibility).

**Figure 3:**
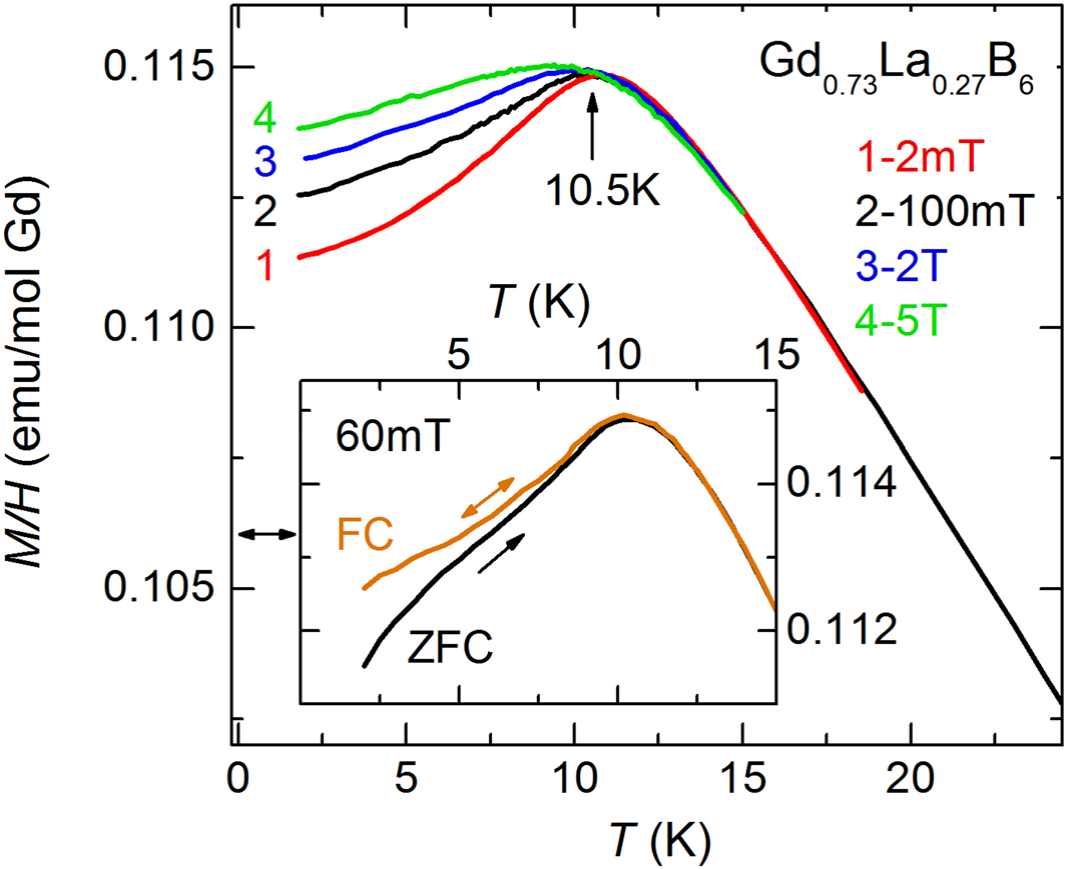
Magnetic susceptibility of Gd_0.73_La_0.27_B_6_ at different fields (inset: FC and ZFC susceptibility).

Magnetic properties of GdB_6_ and Gd_0.73_La_0.27_B_6_ in PM phase look very similar. At high temperatures magnetization of both compounds obeys to Curie–Weiss law *χ* ~ *μ*_*eff*_^2^/(*T* + *Θ*) (insert in Fig. 2). The effective magnetic moment *μ*_*eff*_ ≈ 8.2*μ*_*B*_ derived for GdB_6_ is in agreement with previously published results^34^. The determination of *μ*_*eff*_ for the doped sample strongly depends on the exact actual value of the doping level *x*. In this case we assume that the effective magnetic moment of Gd^3+^ ion in the doped crystal is the same as in GdB_6_ at high temperatures thus obtaining the value *x* = 0.27 which appear in reasonable correlation with the nominal doping. The doping with La leads to the decrease of the Curie–Weiss parameter *Θ* from *Θ* = − 66 K to *Θ* = − 46 K. Note that the value of *Θ* in Gd_0.73_La_0.27_B_6_ is not sensitive to the choice of *x* and remains almost the same if one formally uses the nominal concentration *x* = 0.22. The decline of *Θ* to lower value well correlates with the doping level *x* and is likely resulted from the reduction of the average coordination number *z* for Gd^3+^ spins. The deviation of 1/*χ*(*T*) from the linear behavior caused by short-range correlations is characteristic of both compounds in the intermediate temperature range, although for GdB_6_ it begins at higher temperatures (see the inset in Fig. 2). The drastic difference in magnetic behavior of two samples is seen at low temperatures. The first order phase transition to the antiferromagnetic state observed in GdB_6_ at Neel temperature *T*_*N*_ = 15.5 K is consistent with the previously published data^29^. It appears as a jump on the susceptibility curve *χ*(T) down on ~ 6% at *T*_*N*_ with further gradual decrease of *χ* (Fig. 2). In contrast to GdB_6_, the magnetization of Gd_0.73_La_0.27_B_6_ at low temperatures depends on the sample history. The *M*(*T*) dependencies show smooth highs at *T*_*f*_ ≈ 10.5 K (Fig. 2) and their traces below *T*_*f*_ are defined by the applied magnetic field value (Fig. 3). This behavior may be a consequence of the spin glass (SG) state formation below *T*_*f*_ (*T*_*f*_ in this case is the temperature of spin ensembles freezing) although in “canonical” SGs the magnetization maximum is believed to be sharp at low magnetic fields^35^. In our case the peak shape becomes much sharper with the field decrease but remaining slightly round even at *H* = 20 Oe. However, the magnetization maximum in Gd_0.73_La_0.27_B_6_ can be smeared due to macroscopic inhomogeneity of distribution of La ions in the sample, and further experiments clarifying this question are necessary. The discrepancy between *M*(*T*)/*H* curves begins at one temperature coinciding with the susceptibility maximum thus the further magnetization decrease caused by AFM ordering may be associated with the variety of frozen spin ensembles below *T*_*f*_. Another crucial criteria characterizing the emergence of SG state is the splitting of zero field cooled (ZFC) and field cooled (FC) magnetization curves. The magnetic field *H* = 600 Oe was applied to zero-field cooled sample Gd_0.73_La_0.27_B_6_ at *T* = 2 K, and the sample was heated after that to *T* > *T*_*f*_ (Fig. 3). The magnetization curve in this case continuously tends to the FC curve and reaches it at *T* = *T*_*f*_ . Both the above methods unambiguously testify the onset of SG state in Gd_0.73_La_0.27_B_6_ below *T*_*f*_. It is necessary to note that Gd^3+^ spins concentration is much higher than the magnetic percolation limit and the long range magnetic order would be expected formally for this composition.

**Figure 4:**
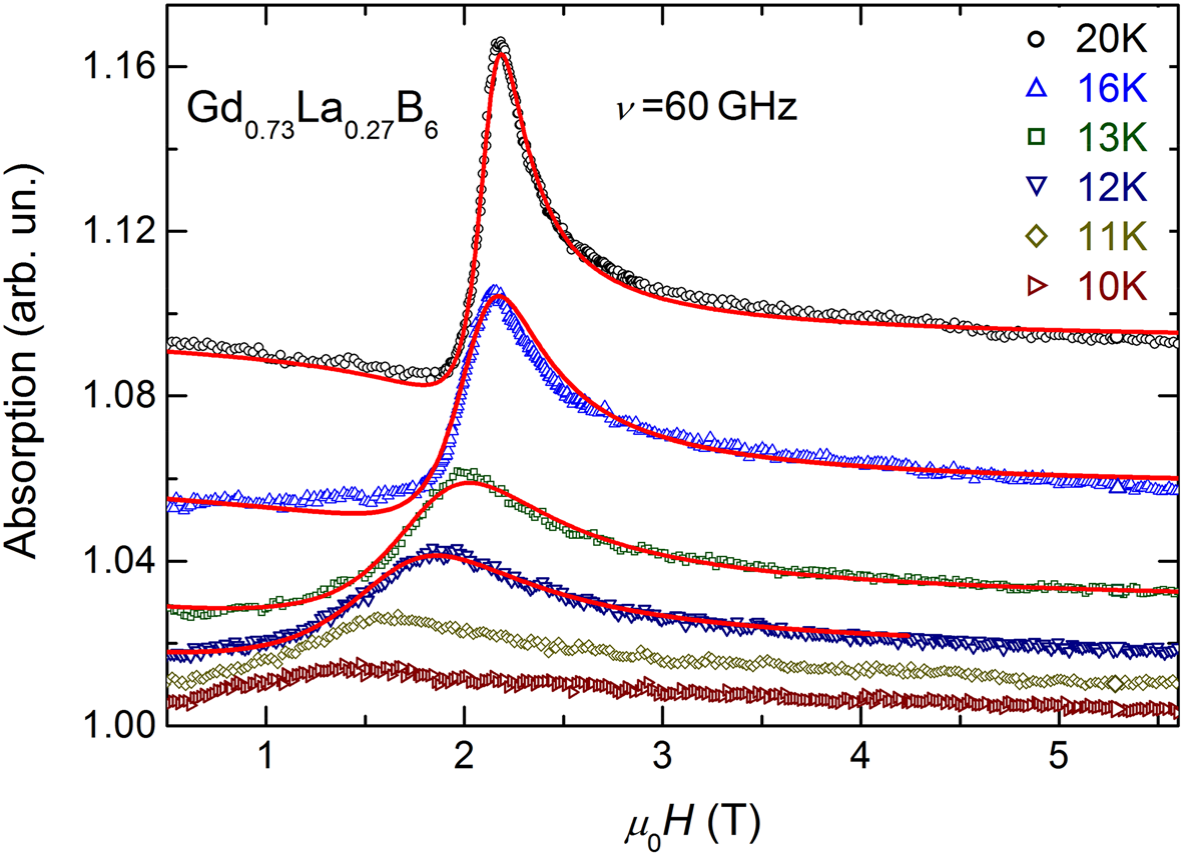
Experimental ESR spectra for Gd_0.73_La_0.27_B_6_ at ν = 60 GHz (open symbols) and the corresponding fits (red solid lines).

**Figure 5:**
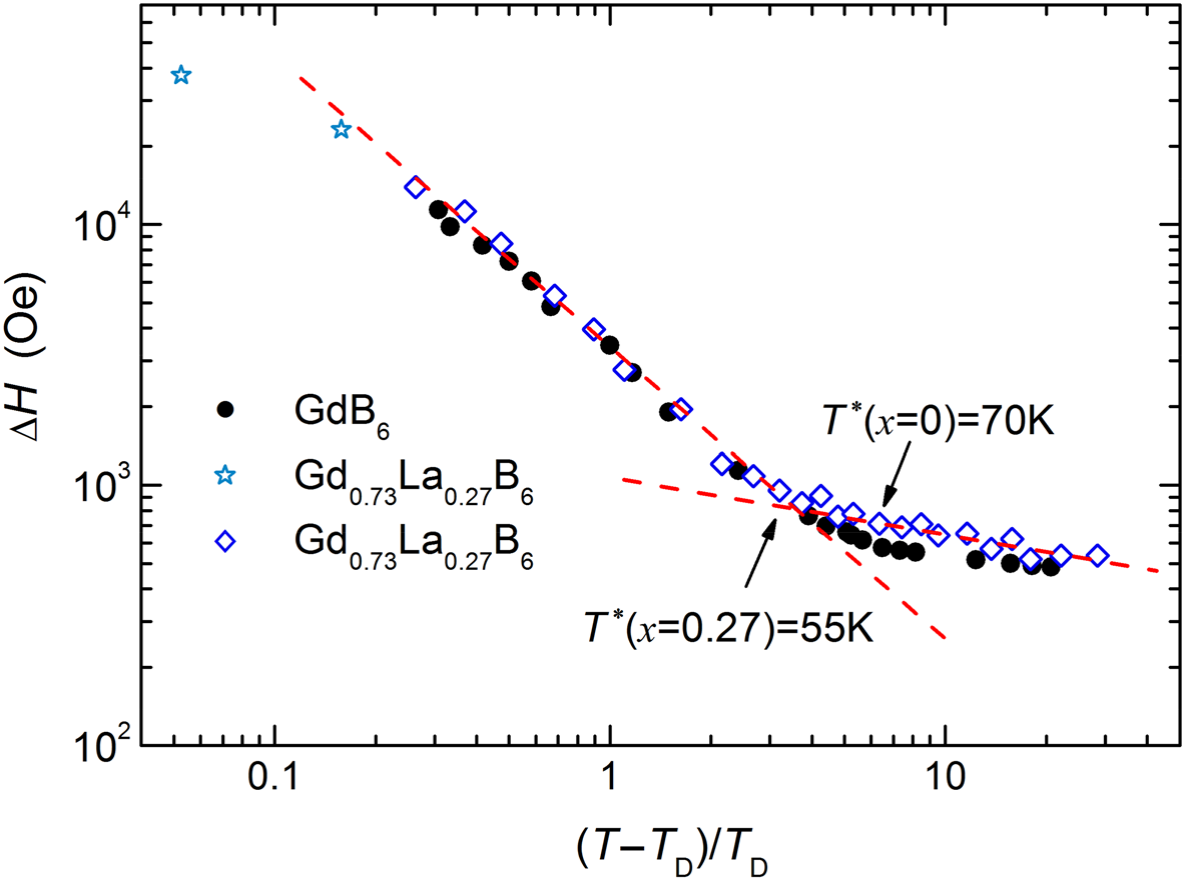
Temperature dependencies of the linewidth of GdB_6_ (closed circles) Gd_0.73_La_0.27_B_6_ (open rhombus and asterisks). For clarity power low fits are shown only for x = 0.27 by dashed lines.

Electron spin resonance (ESR) is the method, which is very useful to investigate spin dynamic in the SG state^36^. It is known from several previous studies that ESR is well observable in the PM phase of GdB_6_ as a single absorption line, which continuously broadens with temperature decrease and its position shifts to lower fields^37–40^. The resonance absorption in the AFM phase was discovered only recently^41^: the resonance line abruptly transforms to the AFM spectrum with complicated behavior at *ν* > 39 GHz, while at lower frequencies the line disappears just below *T*_*N*_^41^. It is remarkable that the line width in PM phase does not diverge down to Neel temperature *T*_*N*_ = 15.5 K. In the PM phase of Gd_0.73_La_0.27_B_6_ ESR line temperature behavior is similar to the case of GdB_6_. However, in the contrast to the parent compound the linewidth diverges at *T*_*D*_ ≈ 9.5 K and no resonance absorption is detected below *T*_*D*_ (Fig. 4). The analysis of the linewidth temperature dependence *ΔH*(*T*) of Gd_0.73_La_0.27_B_6_ demonstrates that it obeys well to the power law: *ΔH*(*T*) ~ ((*T *− *T*_*D*_)*/T*_*D*_)^−*α*^ with *T*_*D*_ = 9.5 K and the exponent *α* = 1.12 ± 0.5 (Fig. 5). It is necessary to remark that ESR linewidth becomes comparable with the resonance field at temperatures *T* ≤ 11 K. Thus, the considerable part of the resonance intensity lies out of the range of magnetic field. At this condition the application the line modeling procedure is no more correct. The *ΔH* parameters for two points in this range evaluated by integration (marked with asterisks in Fig. 4) have underestimated values and were not used in the analysis of *ΔH*(*T*) dependence.

The finding of the power law of *ΔH*(*T*) dependence in Gd_0.73_La_0.27_B_6_ stimulates the looking for the same behavior in GdB_6_ by adjusting the temperature of the width divergence *T*_*D*_ < *T*_*N*_. It is seen that such an analysis results in the coincidence of *ΔH*(*T*) dependencies of both compounds (Fig. 5). The parameters for the *ΔH*(*T*) power law dependence in this case are *T*_*D*_ = 12 K and *α* = 1.09 ± 0.05. Critical dependencies in both systems persist in very wide temperature range: they last up to T* ~ 75 K in GdB_6_ and up to T* ~ 55 K in Gd_0.73_La_0.27_B_6_, where they go into high temperature asymptotic. It is remarkable that the tendency of the line broadening with temperature decrease takes place in both also in the range *T* > T* although the growth is much weaker then at lower temperatures. The *ΔH*(*T*) dependencies can be described by power law in high temperature interval as well with exponents *α* = 0.14 for *x* = 0 and *α* = 0.22 for *x* = 0.27. However it should be born in mind that such an analysis is questionable due to small temperature range of the observation and large temperature separation from *T*_*D*_.

Before discussing the results, it is necessary to examine some important problems regarding the interpretation of ESR experiment. First, the total linewidth may contain, apart from the “critical” term, also additional contributions, which become substantial at temperatures much higher then *T*_*D*_. In the pioneer work of Huber, where the power law was suggested for ESR in SGs, this contribution to the linewidth was described as a temperature independent term *B*^42^. Indeed, this type of high temperature behavior takes place in some of SGs^43^. However, many systems demonstrate either increase or decrease of the linewidth with temperature growth. In the first case the corresponding dependence obeys to Korringa law *a* + *bT*^44^ and it is often observed in metallic alloys^21,45^. The second dependence type is more usual for diluted semiconductors and some frustrated antiferromagnets^46,47^. The analysis of high temperature behavior in this case have shown that *ΔH*(*T*) dependence can be described by semi-empirical function *B*(1 + *Θ*/*T*) where *Θ* is Curie–Weiss parameter^47,48^. Note, that in both latter cases the magnitude of *ΔH* variation at high temperatures may be comparable with its change in the “critical” region. Thus the separation of these two contributions introduces additional uncertainty to the final result.

Another problem is the analysis of ESR line parameters when approaching *T*_*D*_. Due to strong broadening of the line and its shift to lower fields, the considerable part of spectral intensity goes beyond the measuring field range and the data can’t be correctly analyzed by a numerical modeling. The analysis of line shape near *T*_*D*_ can be additionally disturbed due to the deformation of the line shape at the condition when the applied field becomes weaker compared with the random local fields. Then the shape becomes no longer simple, neither Lorentzian nor Gaussian^49^. In this regard, it is necessary to emphasize the importance of increasing the measurement frequency, which allows to expand proportionally the range of observation of the critical dependence.

In the current study the area of critical behavior begins already at temperatures T*/*T*_*D*_ ~ 6–7 where the changeover of the dominant spin relaxation process takes place (Fig. 4). Results of inelastic X-ray scattering experiment show the appearance and further grows of the anharmonicity of free energy potential of Gd^3+^ ions with a decrease in temperature happening somewhere in the range 40–300 K^50^, and the switching between the two types of dependencies of the linewidth at T* occurs apparently for this reason. The use of the high frequency *ν* = 60 GHz in the experiment allowed us to analyze the resonance lines with widths up to *ΔH* ≈ 20 kOe. Due to the above circumstances the range of observation of the critical behavior with *α* ≈ − 1 reached almost two tens, which to our knowledge, considerably exceeds all previous measurements.

Despite the long history of studies of ESR in SGs and the large amount of experimental data obtained, the relationship between the behavior of the resonance line and the properties of the SG state remains an unresolved issue. Generally, SGs can be attributed to the group of systems with short-range magnetic correlations, such as frustrated and low-dimensional magnets, in which the linewidth dependence *ΔH*(*T*) is characterized by strong growth when the temperature decreases toward some critical value *T*_*D*_. According to the most common scenario, *ΔH*(*T*) shows the critical divergence ((*T *− *T*_*D*_)*/T*_*D*_)^−*α*^ , which was observed experimentally and was justified by theoretical models^42^. As for SGs, first observation of the power law of the linewidth with the power exponent *α* = − 1 was reported for MnCu system^51^. It should be noted that this value of *α* is quite common for various systems and is observed both in other SGs^52^ and in frustrated and one-dimensional magnets (*T*_*D*_ equals zero in the latter case)^53^. The experimental and theoretical results supports the opinion that the exponent in SGs should be *α* = − 1^36^. However, in many SGs the value of *α* differs markedly from *α* = − 1^47,54^ and in some semiconductors *α* even varies by almost an order of magnitude depending on the doping level^47^. Thus, numerous experimental results provide a deep basis for considering *ΔH*(*T*) dependence in SGs as power law, however it is not clear how basic line broadening mechanisms affect on the power exponent *α*.

Mention should be made of another approach used to describe the linewidth behavior at low temperatures, which includes the Arrhenius-type dependence of *ΔH*(*T*): *Aexp*(− *T*/*T*_*0*_)^55–57^. Since this model, in contrast to critical behavior, predicts the finite value of *ΔH* at any temperature, the type of dependence can be uniquely determined by careful measurement and analysis of the resonance line near *T*_*D*_.

The observation of the exponent close to *α* = − 1 in GdB_6_ and Gd_0.73_La_0.27_B_6_ is a rather remarkable fact, since it adds one more system with a unique mechanism of the occurrence of short-range correlations to the group of diverse magnets, that demonstrate this type of behavior. This suggests the existence of a common dynamical mechanism for broadening of the resonance line in these systems. In this matter, GdB_6_ is not only one more compound in this set but it can serve as a model system due to the simple crystal structure, the absence of static disorder and the definiteness of the magnetic moment of each cell. One more question posed by current research is the possible difference between the divergence temperature of ESR *T*_*D*_ and the SG temperature *T*_*f*_ observed in the dependence of magnetization in Gd_0.73_La_0.27_B_6_ (*T*_*D*_ = 9.5 K and *T*_*f*_ = 10.5 K). As far as we know, these temperatures were implicitly considered equal in all previous works and the present observation of their discrepancy is the first direct detection of the problem.

It is interesting to consider the features of low-temperature phases in GdB_6_ and Gd_0.73_La_0.27_B_6_ from the point of view of their behavior in the PM phase. The coincidence of *ΔH*(*T*) dependencies testifies the identity of the origin of short-range magnetic correlations in the PM phases of both compounds. Apparently this phenomenon is caused by mutual shifts of Gd^3+^ ions which form dynamical configurations with random distribution of inter-ionic distances and as a consequence of the exchange energy. The ESR in this case can be considered as a sum of resonances from individual Gd^3+^ ions moving in random effective magnetic fields (static and microwave), which cause the line broadening and its shift. Such disordered spin structures freeze in the case of Gd_0.73_La_0.27_B_6_ when the temperature decreases below *T*_*f*_. However, the transition to the AFM state with a coherent ion shift structure in GdB_6_ occurs at higher temperature *T*_*N*_ = 15.5 K thus masking the expected SG temperature *T*_*f*_ = 13.2 K which can be estimated using the relation *T*_*f*_/*T*_*D*_ in Gd_0.73_La_0.27_B_6_. This fact indicates another competitive physical mechanism responsible for the onset of the ordered phase below *T*_*N*_. Indeed, ESR confirms this assumption. According to the experiment, the gyromagnetic ratio *γ* obtained from ESR changes abruptly at *T*_*N*_ and the value *γ* persists at all temperatures in the AFM phase^58^, which signify the onset of new magnetic state of Gd pairs. This effect is similar to the formation of dimers although the magnetic ground state of Gd pairs is different from singlet and its genesis requires a separate study. In its turn, ESR in Gd_0.73_La_0.27_B_6_ doesn’t show any signature of new state of Gd^3+^ emergence. Apparently, the preference of the long-rang order in this compound is destroyed by La doping and the system freeze as a configuration of mutually shifted individual Gd^3+^ ions with various interionic distances. Due to the inhomogenity of La in the sample, one can raise the question of the influence of its distribution on the formation of SG state. According to the above discussion SG transition is qualitatively determined by dynamic of Gd ions matrix, although the doping features can affect as the freezing temperature and the observed difference between *T*_*f*_ and *T*_*D*_.

**Figure 6:**
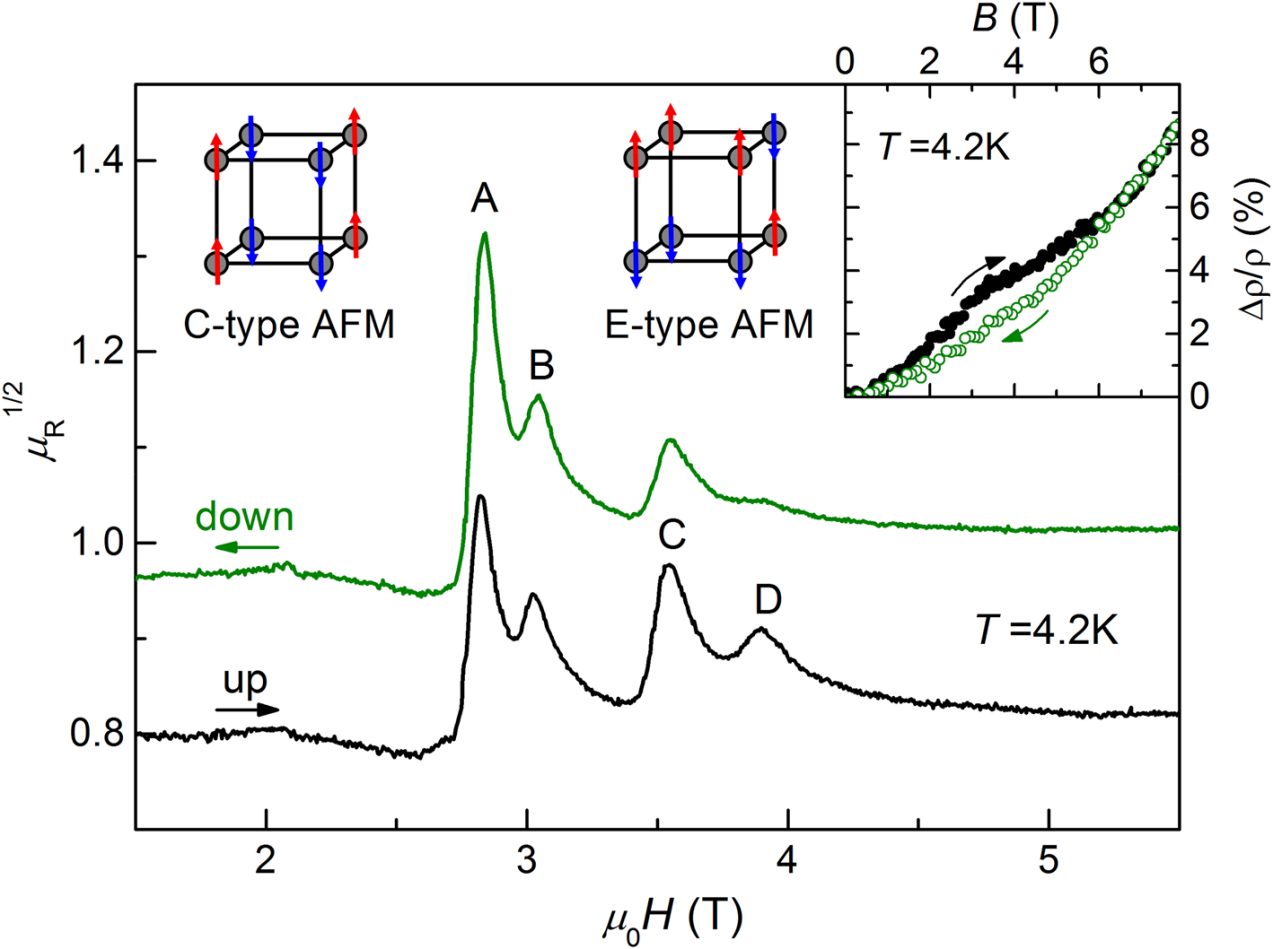
Hysteretic behavior of ESR in AFM2 phase of GdB_6_. Two types of possible magnetic structures are illustrated. The inset shows the hysteresis in magnetoresistance^33^.

Based on the above consideration, it is worth paying attention to the low temperature range (*T* < 10–12 K) inside the AFM phase of GdB_6_ where a new state of AFM2 develops. Note that in spite of several X-ray studies^23–26^ the displacement structure in this area is not recognized^26^. The anomalous features of AFM2 phase are the hysteretic behavior of some physical parameters as well as the sample dependence of this effect. So, the hysteresis was observed in the resistivity and magnetization at *T*_*N*2_ in some experiments^29,59^ although it is absent in some other samples^33^. The dependence, which is known to exhibit hysteresis in all studied samples, is magnetoresistance^33,59^. Moreover, the value *T*_*N2*_ varies in the range 5–12 K in different experiments^23–26,29,33,59^. In the recent density functional calculations of GdB_6_ the basic structural and electronic properties as well as the stability of different AFM structures were determined^60^. It turns out that two types of magnetic orders, E-AFM and C-AFM (illustrated in Fig. 6), lay energetically close to each other in the wide range of the Coulomb repulsion parameter *U* with energy difference 0.6–1 meV (6–12 K) between them^60^. In its turn, the hysteretic properties of ESR absorption suggest the coexistence in AFM2 phase of domains with different types of magnetic structure. Four lines resonance structure develops below *T* ≤ 12 K^41^ and the resonance “sweep up” spectra become different from that “sweep down” ones in the range 4.2 K ≤ *T* < 10 K . (Fig. 6). This is reflected in the redistribution of the sum absorption intensity between different resonance lines, while the total intensity of integral spectrum remains constant. It was assumed in the previous study that ESR spectrum in AFM phase is determined by low-symmetrical crystal field arising due to the shift of Gd^3+^ ions^41^. However, the change in line intensity can hardly be explained within the framework of this hypothesis. Indeed, any change of crystal field parameters would rather affect lines positions but not exclusively the distribution of intensities. On the other hand, this observation is consistent with the assumption that the AFM phase consists of domains with different magnetic orders. Moreover, proportional change of the lines A and B as well as C and D (Fig. 6) suggests that each pair of lines belongs to different domains. At the same time, the arrangement of pairs in each structure is clearly defined, as evidenced by the small width of the resonance line, and also slightly different from each other. Note, that the magnetic structure one of two lowest states E-AFM is consistent with neutron experiment results^27,28,60^. The absence of the satellites of the second structure may be caused by small volume of the corresponding domain as well as the complexity of the neutron experiment in GdB_6_^27,28^. The considerable difference in domains volumes can be seen from the difference of intensities of the corresponding lines, which takes place at all frequencies^41^.

The competition of two magnetic structures can explain the puzzling feature of AFM2 phase which lies in the fact that hysteresis is observed in magnetoresistance and ESR (Fig. 6)^33^, while is not visible in magnetization field dependence. Apparently, the magnetization at low temperatures is determined by the exchange energy in Gd pairs characterized by the exchange field *H*_*E*_ ≈ 245 kOe^61^, and one can assume that this parameter in the magnetic structures of close energy is also close. Then the volume redistribution between domains of different type will not noticeably affect the sum magnetization. In its turn, the position of resonance line is determined by the parameter (*H*_*A*_*H*_*E*_)^1/2^, which include the anisotropy field *H*_*A*_ as well. Thus, possible variations in *H*_*A*_ in different positions of pairs can change the position of the lines without a detectable effect on the magnetization. The redistribution of Gd pairs between different positions in this case affects not only on ESR mode intensities but also on resistivity via the change of the configuration of scattering array consisting of nonequivalent Gd pairs thus causing its hysteresis.

Within the hypothesis of the coexistence of domains with two magnetic structures the transition between AFM2 and AFM1 phases inside AFM phase looks like gradual “mixing” of stable domains when temperature increases. So, the AFM1 phase consists from random dynamical complexes of Gd ions. However, in contrast to the PM phase, the structural element of it is Gd pair and possible configurations are restricted by certain combinations of E-AFM and C-AFM clusters. The structure of AFM1 phase can be described within the framework of a single state as shift wave of Gd ions^26^. Disturbance of positions and arrangements of Gd pairs lead to strong broadening of ESR line in this phase. However, the resonance line exactly cover the position of the spectrum of A,B,C,D lines thus confirming the lack of magnetic structures except those existing in AFM2 phase. Therefore it is possible to assume that multi-line ESR spectrum structure is caused by presence of several types of domains. In its turn, the domains formation may be expected to be very sensitive to the minor intrinsic defects and impurities of the crystal structure which would explain the large spreading of *T*_*N2*_ value found in the experiment.

In the connection of the present study, it is necessary to mention the rich borides, where the possibility of the glass behavior caused by the displacement of RE ions from their position in the lattice is discussed in literature. The signs of SG behavior were found in PrB_6_^62,63^ and the presence of the “cage-glass” state was claimed taking place in dodecaborides LuB_12_ and ZrB_12_^64,65^. However, as for magnetic and nonmagnetic RE ions the origin of the assumed glass effects is inherent defects in the boron lattice which induce the ions shift. Moreover the estimated defect concentration in these compounds is rather small (< 5%)^66^ and the existence of the volume glass states remains the disputable question^67^. In this respect GdB_6_ and Gd_0.73_La_0.27_B_6_ are first systems where the origin of SG behavior is not caused by the inherent disorder in the magnetic Gd^3+^ ion system but is induced by ions shift with the formation of random spin configurations. It leads to short range spin correlations in the PM phase and then SG freezing in Gd_0.73_La_0.27_B_6_ or SG effects in AFM2 phase of GdB_6_ with temperature lowering. It should be emphasized that doping with La is not a source of the SG state in the system but rather leads to suppression of competitive coherent ordering with simultaneous decrease in *T*_*f*_.

In conclusion, the magnetization measurements of Gd_0.73_La_0.27_B_6_ have shown the onset of SG state below *T*_*f*_ = 10.5 K. It manifests itself by the maximum on *M*(*T*) at *T*_*f*_ and by the dependence of *M* on the sample history below *T*_*f*_. The identity of the temperature dependencies of ESR linewidth in the PM phase of Gd_0.73_La_0.27_B_6_ and GdB_6_, which follow the power law ((*T *− *T*_*D*_)/*T*_*D*_)^−α^ with *α* ≈ − 1, clearly demonstrate the SG origin of short range magnetic correlations underlying the line broadening. In the case of Gd_0.73_La_0.27_B_6_ it leads to the width divergence at *T*_*D*_ = 9.5 K while in GdB_6_ the coherent AFM phase transition takes place at *T*_*N*_ = 15.5 K hiding the related SG temperature *T*_*D*_ = 12 K. The observed behavior is caused by the shift of Gd^3+^ ions from the centrally symmetrical positions in the rigid boron lattice. In Gd_0.73_La_0.27_B_6_ dynamical displacement complexes get frozen at *T*_*f*_ resulting to the SG phase. The coherent displacement of Gd ions compete in GdB_6_ with random configurations leading first to first order phase transition and then at *T* < *T*_*D*_ to the onset of complicated low temperature phase with peculiar hysteretic behavior.

## Methods

The single crystals of GdB_6_ and of Gd_1−x_La_x_B_6_ (with nominal composition *x* = 0.22) were grown by the induction zone melting in argon atmosphere. The sample of GdB_6_ is identical to those ones studied previously in transport (Fig. 1b) and ESR measurements^33,41^. The quality of both crystals is verified by the X-ray diffraction technique, microprobe analysis and SEM. The latter method did not allow the exact actual concentration of La to be measured due to inhomogeneous distribution of the dopant with a spread of *x* of several percent. The ESR measurements have been done using the setup based on Agilent PNA network analyzer^68^. Method for cavity measurements of strongly correlated metals, where samples are fixed as a part of the bottom plate of the cylindrical cavity^69–72^ was applied. Experiments were carried out in cylindrical cavity operating on TE_011_ mode at the frequency *ν* = 60 GHz. The magnetic field was applied along [100] crystallographic direction in both cases. The experimental resonance curves were analyzed as the sum of real *χ*_1_(*H*) and imaginary *χ*_2_(*H*) parts of microwave magnetic susceptibility where *χ*_1_(*H*) and *χ*_2_(*H*) were taken as Lorentz functions, except in the case of very wide lines that is discussed in the text. Magnetic measurements have been carried out with the help of SQUID magnetometer MPMS-5 (Quantum Design) at fields up to 5 T.

## References

[1] Süllow S (1997). Spin glass behavior in URh_2_Ge_2_. Phys. Rev. Lett..

[2] Gardner JS, Gingras MJP, Greedan JE (2010). Magnetic pyrochlore oxides. Rev. Mod. Phys..

[3] Booth CH, Han SW, Süllow S, Mydosh JA (2004). Local lattice symmetry of spin-glass and antiferromagnetic URh_2_Ge_2_. J. Magn. Magn. Mater..

[4] Süllow S (2000). Disorder to order transition in the magnetic and electronic properties of URh_2_Ge_2_. Phys. Rev. B.

[5] Nishioka T, Tabata Y, Taniguchi T, Miyako Y (2000). Canonical spin glass behavior in Ce_2_AgIn_3_. J. Phys. Soc. Jpn..

[6] Tien C, Feng CH, Shui C, Lu JJ (2000). Ce_2_CuGe_3_: A nonmagnetic atom-disorder spin glass. Phys. Rev. B.

[7] Li DX, Kimura A, Haga Y, Nimori S, Shikama T (2011). Magnetic anisotropy and spin-glass behavior in single crystalline U_2_PdSi_3_. J. Phys. Condens. Matter.

[8] Szlawska M, Gnida D, Kaczorowski D (2011). Magnetic and electrical transport behavior in the crystallographically disordered compound U_2_CoSi_3_. Phys. Rev. B.

[9] Li, D., Homma, Y., Honda, F., Yamamura, T. & Aoki, D. Low temperature spin-glass behavior in nonmagnetic atom disorder compound Pr_2_CuIn_3_. In *Physics Procedia 75. 20th International Conference on Magnetism, ICM2015* 703–710 (2015).

[10] Krimmel A (1999). Spin-glass behavior in PrAu_2_Si_2_. Phys. Rev. B.

[11] Anand VK, Hossain Z, Adroja DT, Geibel C (2011). Signatures of spin-glass behaviour in PrIr_2_B_2_ and heavy fermion behaviour in PrIr_2_B_2_. J. Phys. Condens. Matter.

[12] Anand VK, Adroja DT, Hillier AD, Taylor J, Andre G (2011). Signatures of spin-glass behavior in the induced magnetic moment system PrRuSi_3_. Phys. Rev. B.

[13] Anand VK, Adroja DT, Hillier AD (2012). Ferromagnetic cluster spin-glass behavior in PrRhSn_3_. Phys. Rev. B.

[14] Górnicka K, Kolincio KK, Klimczuk T (2018). Spin-glass behavior in a binary Pr_3_Ir intermetallic compound. Intermetallics.

[15] Goremychkin EA (2008). Spin-glass order induced by dynamic frustration. Nat. Phys..

[16] Gaulinx BD, Reimers JN, Mason TE, Greedan JE, Tun Z (1992). Spin freezing in the geometrically frustrated pyrochlore antiferromagnet Tb_2_Mo_2_O_7_. Phys. Rev. Lett..

[17] Zhou HD, Wiebe CR, Harter A, Dalal NS, Gardner JS (2008). Unconventional spin glass behavior in the cubic pyrochlore Mn_2_Sb_2_O_7_. J. Phys. Condens. Matter.

[18] Thygesen PMM (2017). Orbital dimer model for the spin-glass state in Y_2_Mo_2_O_7_. Phys. Rev. Lett..

[19] Kasuya T (1997). Exchange-pair Jahn–Teller effects in GdB_6_. J. Magn. Magn. Mater..

[20] Takahashi Y, Ohshima K, Okamura F, Otani S, Tanaka T (1999). Crystallographic parameters of atoms in the single crystals of the compounds RB_6_ (R = Y, La, Ce, Nd, Sm, Eu, Gd). J. Phys. Soc. Jpn..

[21] Taylor RH, Coles BR (1975). Electron spin resonance studies of the onset of magnetic order in intermetallic compounds. J. Phys. F Met. Phys..

[22] Iwasa K (2011). Motion of the guest ion as precursor to the first-order phase transition in the cage system GdB_6_. Phys. Rev. B.

[23] Galéra R-M, Osterman DP, Axe JD (1988). X-ray scattering study of the magnetic phase transformation in GdB_6_. J. Appl. Phys..

[24] Kuwahara K (2005). Resonant and non-resonant X-ray scattering from GdB_6_. Phys. B.

[25] McMorrow DF (2004). Coupling of lattice and spin degrees of freedom in GdB_6_. Phys. B.

[26] Amara M (2005). Exchange-displacement waves in GdB_6_. Phys. Rev. B.

[27] Kuwahara K (2002). EXCED—Epithermal neutron diffractometer at KENS. Appl. Phys. A.

[28] Luca S (2004). Neutron diffraction studies on GdB_6_ and TbB_6_ powders. Phys. B.

[29] Nozaki H, Tanaka T, Ishizawa Y (1980). Magnetic behaviour and structure change of GdB_6_ single crystals at low temperatures. J. Phys. C Sol. St. Phys..

[30] Tanaka T, Nishitani R, Oshima C, Bannai E, Kawai S (1980). The preparation and properties of CeB_6_, SmB_6_, and GdB_6_. J. Appl. Phys..

[31] Kunii S (1985). Electronic and magnetic properties of GdB_6_. J. Magn. Magn. Mat..

[32] Reiffers M, Šebek J, Šantavá E, Pristáš G, Kunii S (2006). Thermal hysteresis of the phase-transition temperature of single-crystal GdB_6_. Phys. Stat. Sol. (b).

[33] Anisimov M (2017). Anisotropy of the charge transport in GdB_6_. Acta. Phys. Pol. A.

[34] Coles BR, Griffiths D (1961). Antiferromagnetic behaviour of GdB_6_. Proc. Phys. Soc..

[35] Midosh JA (2015). Spin glasses: Redux: An updated experimental/materials survey. Rep. Prog. Phys..

[36] Huang CY (1985). Some experimental aspects of spin glasses: A review. J. Magn. Magn. Mat..

[37] Coles BR, Cole T, Lambe J, Laurance N (1962). Electrical resistivity and paramagnetic resonance in gadolinium hexaboride. Proc. Phys. Soc..

[38] Fisk Z, Taylor RH, Coles BR (1971). Anomalous magnetic behaviour of gadolinium borides. J. Phys. C Solid State Phys..

[39] Miller DE, Hacker H (1971). Paramagnetic resonance of GdB_6_. Sol. St. Commun..

[40] Sperlich G, Janneck KH, Buschow KHJ (1973). Exchange narrowing in the ESR spectra of metallic Gd_x_La_1–x_B_6_ (x = 1 to 0.01). Phys. Stat. Sol. (b).

[41] Semeno AV (2018). Antiferromagnetic resonance in GdB_6_. JETP Lett..

[42] Huber DL (1972). Critical-point anomalies in the electron-paramagnetic-resonance linewidth and in the zero-field relaxation time of antiferromagnets. Phys. Rev. B.

[43] Monod P, Landi A, Blanchard C, Deville A, Hurdequint H (1986). Paramagnetic linewidth analysis of ESR in spin glasses. J. Magn. Magn. Mater..

[44] Barnes SE (1981). Theory of electron spin resonance of magnetic ions in metals. Adv. Phys..

[45] Zomack M, Baberschke K, Barnes SE (1983). Magnetic resonance in the spin-glass (LaGd)Al_2_. Phys. Rev. B.

[46] Jamet JP, Dumais JC, Seiden J, Knorr K (1980). Magnetic resonance of Mn^2+^ in an amorphous spin-glass insulating manganese aluminosilicate (Mn_3_Al_2_Si_3_O_12_). J. Magn. Magn. Mater..

[47] Oseroff SB (1982). Magnetic susceptibility and EPR measurements in concentrated spin-glasses: Cd_1−x_Mn_x_Te and Cd_1−x_Mn_x_Se. Phys. Rev. B.

[48] Dormann E, Jaccarino V (1974). High temperature EPR linewidths in MnO and MnS. Phys. Lett. A.

[49] Kubo, R. & Toyabe, T. In *Magnetic Resonance and Relaxation* (ed. Blink, R.) 810 (North-Holland, Amsterdam, 1967).

[50] Iwasa K (2014). Universality of anharmonic motion of heavy rare-earth atoms in hexaborides. J. Phys. Soc. Jpn..

[51] Salamon MB, Herman RM (1978). Critical dynamics and spin relaxation in a Cu–Mn spin-glass. Phys. Rev. Lett..

[52] Salamon MB (1979). Freezing of EPR relaxation rates in spin glasses. Solid State Commun..

[53] Oshikawa M, Affleck I (1999). Low-temperature electron spin resonance theory for half-integer spin antiferromagnetic chains. Phys. Rev. Lett..

[54] Malozemoff AP, Krusin-Elbaum L, Taylor RC (1981). Spin resonance and magnetic susceptibility of amorphous Gd_x_Y_0.33−x_Al_0.67_ films showing spin-glass behavior. J. Appl. Phys..

[55] Webb DJ, Bhagat SM, Furdyna JK (1984). Electron paramagnetic resonance linewidths in diluted magnetic semiconductors: Cd_1−x_Mn_x_Te. J. Appl. Phys..

[56] Sayad HA, Bhagat SM (1985). Dynamic random fields in diluted magnetic semiconductors: Cd_1−x_Mn_x_Te. Phys. Rev. B.

[57] Mantilla JC (2005). Magnetic resonance in the Zn_1−x_Mn_x_In_2_Se_4_ dilute magnetic semiconductor system. J. Phys. Condens. Matter.

[58] Semeno AV, Gilmanov MI, Shitsevalova NYu, Demishev SV (2019). Properties of antiferromagnetic resonance in GdB_6_. J. Phys: Conf. Ser..

[59] Ali N (1988). Anomalous electrical and magnetic properties of gadolinium hexaboride. J. Appl. Phys..

[60] Xu S (2019). Interplay of electronic, magnetic, and structural properties of GdB_6_ from first principles. Phys. Rev. B.

[61] Sugiyama K, Koyoshi Y, Kunii S, Kasuya T, Date M (1988). High field magnetization of GdB_6_. J. Phys. Soc. Jpn..

[62] Alekseev PA (2010). Specific features of the formation of the ground state in PrB_6_. Phys. Sol. St..

[63] Anisimov MA (2012). Suppression of spin-glass state in PrB_6_. Solid State Phenom..

[64] Sluchanko NE (2011). Effects of disorder and isotopic substitution in the specific heat and Raman scattering in LuB_12_. JETP.

[65] Sluchanko NE (2016). Raman scattering in ZrB_12_ cage glass. JETP Lett..

[66] Menushenkov AP (2013). Features of the local structure of rare-earth dodecaborides RB_12_ (R = Ho, Er, Tm, Yb, Lu). JETP Lett..

[67] Ponosov YuS, Streltsov SV, Levchenko AV, Filippov VB (2016). Electronic Raman scattering and the renormalization of the electron spectrum in LuB_12_. JETP.

[68] Samarin AN (2015). High frequency electron spin resonance in Mn_1-x_Fe_x_Si. Phys. Proc..

[69] Semeno AV (2009). Electron spin resonance in EuB_6_. Phys. Rev. B.

[70] Demishev SV (2009). Magnetic spin resonance in CeB_6_. Phys. Rev. B.

[71] Demishev SV (2018). Magnetic resonance probing of ground state in the mixed valence correlated topological insulator SmB_6_. Sci. Rep..

[72] Demishev SV (2020). Electron spin resonance in strongly correlated metals. Appl. Magn. Reson..

